# The Relationship between Maternal Employment Status and Pregnancy Outcomes

**DOI:** 10.5539/gjhs.v8n9p37

**Published:** 2015-10-16

**Authors:** Farnoush Khojasteh, Azizollah Arbabisarjou, Tahereh Boryri, Amneh Safarzadeh, Mohammad Pourkahkhaei

**Affiliations:** 1 Pregnancy Health Research Center, Zahedan University of Medical Sciences, Zahedan, Iran; 2 Student Scientific Research Center, Zahedan University of Medical Sciences, Zahedan, Iran

**Keywords:** high risks, maternal employment status, pregnancy outcomes

## Abstract

**Background and Objectives:**

Women comprise a large percentage of the workforce in industrial countries. In Europe and many other places in the world, women of reproductive age comprise a significant proportion of the workforce at the workplaces, and the rules and regulations require employers to evaluate and minimize health risks to pregnant women. In U.K, 70%, and in the United States 59% of women are employed. In Iran, 13% of women are employed, which comes down to less than 5% at Sistan& Baluchestan Province. Various studies have reported contradictory results about the effects of maternal employment tasks such as standing, repetitive bending, climbing stairs, and lifting heavy objects during pregnancy on fetal growth, preterm birth and other obstetric complications. Given the growing number of working women, and potential complications for mothers, the present study has conducted to investigate the relationship between maternal employment status and pregnancy outcomes in Zahedan city, Iran.

**Materials and Methods:**

This cross-sectional study was based on survey conducted on 227 women (121 housewives, and 106 employed women) attending health centers in 2014. Using purposive convenient sampling method, eligible pregnant mothers (with no chronic diseases, singleton pregnancy, gravida 1-3, and no addiction) were selected as study subjects. Data were collected and recorded through a researcher-made questionnaire and also from mothers’ medical records, including personal details, prenatal and labor complications, and infant’s details. Collected data were fed into the SPSS version 21(IBM Corp, USA).

**Results:**

Frequency of placental abruption was greater among housewives (P=0.02), and a significant relationship was found between employment status and lifting heavy objects, which was more frequent among housewives (P=0.01). Lifting heavy objects during pregnancy was only significantly related to reduced amniotic fluid (P=0.001) and low birth weight (P=0.01). Frequency of preterm labor was higher among housewives compared to employed women, but not significantly. Type of delivery was significantly related to employment, and employed mothers had more cesarean deliveries (P=0.0001).

**Conclusion:**

The results suggest more frequent lifting of heavy objects by housewives than by employed mothers, leading to increased complications such as reduced amniotic fluid, placental abruption, and low birth weight. Perhaps due to higher education levels, frequency of cesarean section and preterm labor was higher among employed mothers. However, employment alone does not predict pregnancy outcomes.

## 1. Introduction

The need for skilled workers increases with advancement of technology and development of industries. Men cannot be expected to shoulder this growing need alone, and women also carry a significant share of the burden. Studies conducted in recent decades have showed that women comprise half the world’s population, and perform two thirds of productive work worldwide, but only a third of the work they do is formally recognized. Women comprise half of the country population and then it is important to be awared of their condition in different dimensions and planning to amend their condition after the society and family health (Kiani, & Arbabisarjou, 2013). Women comprise a large percentage of the workforce in industrial countries. In Europe and many other places in the world, women of reproductive age comprise a significant proportion of the workforce, and the law requires employers to evaluate and minimize health risks to pregnant women. In England, 70%, and in the United States 59% of women are employed. In Iran, 13% of women are employed, which comes down to less than 5% in Sistan and Baluchestan Province (Kadhodai, 2011; M, 2007).

The rapid increase in population of working women over the past 20 years requires assessment of the effect of employment on reproduction. Workers of 20 million jobs are exposed to reproductive risks, mostly latent, with effects emerging years later. Clearly, pregnant women and their fetuses will not be immune to such complications. This was recognized several centuries ago, but physical activities that might be harmful for fetal health are still not properly known (Jahromi, 2011; Takito, 2010; Arbabisarjou et al., 2011). Physical activities at work might adversely impact on pregnancy outcomes such as preterm delivery and low birth weight (Bonzini, Coggon, & Palmer, 2006; Fortier, Marcoux, & Brisson, 1995; Hanke, Saurel-Cubizolles, Sobala, & Kalinka, 2001; Naseriasl, Pourreza, Akbari, & Rahimi, 2014; Niedhammer, O’Mahony, Daly, Morrison, & Kelleher, 2009; Palmer K, 2013; Xu et al., 2014). Paying attention to physical, mental, social, and cultural health in any society and preparing the conditions for the realization of a dynamic and healthy life guarantee communal health for the future of that society. To achieve this worthy goal, the prevention of emotional disorders, anxiety and depression is essential (Robabi, Arbabisarjou, & Zareban, 2015). Various studies have demonstrated the relationship between materials that working mothers are exposed to and the prevalence of low birth weight, reinforcing the belief that even inside the uterus, the fetus is not safe from materials mothers are exposed to during work (Fortier et al., 1995; Weinraub, Jaeger, & Hoffman, 1988; Xu et al., 2014). A study investigated the effect of noise at work and temperature changes on the course of pregnancy, and showed lower mean birth weight compared to the control group with noise levels in excess of 90 db, especially in women working in one position for long periods of time and with prolonged exposure to excessive noise levels, which is considered a risk factor (Hartikainen, Sorri, Anttonen, Tuimala & Läärä, 1994). The present study was conducted to investigate the effect of mother’s employment on pregnancy outcome in Zahedan city in 2014.

## 2. Materials and Methods

This was a cross-sectional study using cluster sampling. Purposive sampling was conducted during spring and summer 2014 to recruit, all women including housewives who presented to health centers. First, Zahedan was divided into 5 areas (north, south, east, west, and center), and one health center with the most referrals was selected from each area, and the required subjects were selected from these centers for working (106 women) and housewife (121 women) groups. The ethics Committee of Zahedan University of Medical Sciences approved of the study (code:6155) Subjects were conveniently selected from eligible women attending these health centers 6 months after childbirth when they came for vaccination of their children or further examinations. Study inclusion criteria were gravida 1-3 with the first trimester ultrasound results, no systemic or chronic diseases, and singleton pregnancy. Data were collected and recorded through a researcher-made questionnaire.

Mothers’ records included information on age, education level, employment status, type of employment, number of working hours per day (<8, >8), number of hours standing per day, (<8, >8) lifting heavy objects each day. Complications during pregnancy and delivery included placenta abruption, placenta previa, preterm labor, type of delivery, reduced amniotic fluid. In addition, the birth weight right after delivery was measured and recorded in the patient’s files and were collected. The gestational age was estimated based on the first trimester ultrasound. The diagnosis of rupture of membrane was based on patient history, Fern test, and vaginal examination.

The limitations of this research were the relationship of employment status and lifting heaving objects with variables such as placental abruption, low birth weight (LBW) due to small sample size, as a result the results cannot be generalized to the society and a larger sample size is required. Collected data were fed into the SPSS version 21 (IBM Corp, USA) using Chi-square test, paired t test. Results are presented as tables.

Zahedan is a city in Sistan& Baluchestan Province, close to borders of Pakistan and Afghanistan. Its climate is hot and dry although the Tafton extinct volcano is about 100 kilometers away from it. The population of the city is 5.527.060 and people do different things to earn a living for example some are shopkeeper and some are laborers. In the past decades, they educate their children hoping to find jobs in public organizations. Most women are housewives and do chores in their houses. Traditionally, men do not do things at home; they only work out to provide their families with food and clothes (Baloch, 2006; Wikipedia, 2006). Sistan and Balouchestan Province has specific cultural-ethnographical conditions such as tendency to have more children and masculine baby which affect general health of women (Kiani, & Arbabisarjou, 2011).

## 3. Results

According to the results, participants’ mean age was 28.67 years (youngest 15, and oldest 42 years of age). Of the total subjects, 2.6% were illiterate, 12.3% had elementary school and junior high school education, 32.6% had high school education and associate diploma, and 52.4% had higher education. In terms of employment, 46.7% were employed, and 53.3% were housewives. In terms of type of employment, 38.7% were teachers, 18.9% worked in hospitals, and 42.5% were office workers. Mean number of daily working hours was 8.48 (minimum 1 hour and maximum 20 hours). Mean number of standing hours per day was 4.64 (minimum 1 hour and maximum 20 hours). Mean birth weight was 3100 grams (minimum weight 1700 grams, and maximum 4500 grams). The results showed that 2.6% of infants had LBW, 19.6% of deliveries were preterm, 48.5% of deliveries were vaginal, and 51.5% were cesarean section, 0.09% had placenta previa, 0.9% had reduced amniotic fluid, 2.6% had placenta abruption, and 15% of pregnant women had lifted heavy objects. There were no significant differences among pregnant women mean daily working hours and standing hours, in terms of placental abruption, placenta previa, and reduced amniotic fluid, gestational age, or type of delivery. However, frequency distribution of cesarean section was 47.6% in women with less than 8 hours standing per day and 100% in those with more than 8 hours standing per day. A significant relationship was observed between education level and lifting heavy objects (P<0.0001, d.f=3). 93.4% of employed women and only 16.5% of housewives had higher education, and most housewives were either illiterate or had elementary school and junior high school education. A significant relationship was found between lifting heavy objects and employment status (P<0.01, d.f=1), and 8.5% of employed women and 20.7% of housewives lifted heavy objects every day during pregnancy.

No relationship was found between employment status and variables placenta previa, reduced amniotic fluid, or low birth weight. However, there was a significant relationship between employment status and placental abruption (P<0.02, d.f=1), and prevalence of placental abruption was 41.32% among housewives, and women 0% among employed (Table 1).

**Table 1:** Frequency distribution of pregnant women according to employment status and placenta abruptive

Placenta abruption/employment	Placenta abruption		No placenta abruption		Total	
		
	Number	%	Number	%	Number	%
Employed	0	0	106	100	106	100
Housewife	6	5	115	95	121	100
Total	6	2.6	221	97.4	227	100

p<0.02.

There was also a significant relationship between employment status and type of delivery (P<0.0001, d.f=1), 65.1% of employed women and only 39.7% of housewives had cesarean deliveries. Frequency distribution of employment status in terms of gestational age showed 24.8% of employed women and 15.1% of housewives had preterm deliveries, but this was not significant. No significant relationship was found between lifting heavy objects, placenta abruption, placenta previa, type of delivery, or gestational age. There was a significant relationship between lifting heavy objects and loss of amniotic fluid (P<0.001, d.f=1) (Table 2), and loss of amniotic fluid was only seen in 5.9% of women who had lifted heavy objects, but not in others. A significant relationship was also observed between lifting heavy objects and low birth weight (P<0.01, d.f=1), and low birth weight was seen in 8.8% of women who had lifted heavy objects, and 1.6% in others (Table 2).

**Table 2:** Frequency distribution of pregnant women according to lifting heavy objects and low birth weight (below 2500 gram) and reduced amniotic fluid

Lifting Heavy objects	Reduced amniotic fluid					LBW					
	
		Yes		NO		<2500		>2500		TOTAL	
		Number	%	Number	%	Number	%	Number	%	Number	%
	Lifting	2	5.9	32	94.1	2	8.8	3	91.2	34	100
	NO	0	0	193	100	3	1.6	190	98.4	193	100
	Lifting										
	TOTAL	2	9	225	99.1	6	2.6	221	97.4	227	100
	
		P<.01				P<.001					

## 4. Discussion

The results were analyzed in terms of objectives. According to results, there was no significant relationship between employment status and placenta previa, reduced amniotic fluid, or low birth weight. However, most studies have found a significant relationship between physical work and low birth weight, and risk of LBW was greater in women who constantly stood in one position for more than 6 hours per day (Bonzini et al., 2006; M, 2007; Naseriasl et al., 2013; Niedhammer et al., 2009; Palmer, 2013; Weinraub et al., 1988; Xu et al., 2014), which disagrees with the present study results that showed no relationship between LBW and employment status. In the present study, the relationship between employment and placenta abruption was significant. No study was found in this area, though. The increase in placental abruption may be due to the fact that housewives in this study were less educated and took care of themselves less during pregnancy, and performed heavy tasks more frequently, and thus had more placenta abruption and other complications than working women who were more educated and worked in hospitals and education, and looked after themselves better. In the present study, there was a significant relationship between employment status and gestational age, and employed women had more cesarean and preterm deliveries, probably because employed women are higher educated, marry later, and have greater tendency toward elective cesarean and rapid end to pregnancy. This agrees with the results from other studies that found long working hours can cause preterm delivery (Fortier et al., 1995; Niedhammer et al., 2009; Palmer K., 2013; Xu et al., 2014). A Study in Ardebil showed that cesarean section rates were significantly correlated with employment of women, which agrees with our study (Naseriasl et al., 2014). The present study showed the risk of miscarriage increased with working night shifts and frequent staying up late. We reduced this risk by food supplement, regular physical activities and labor health but in this study miscarriage was not a variable (Xu et al., 2014).

In the present study, lifting heavy objects was more prevalent among housewives, which agrees with the results of another study in Iran that showed more undesirable pregnancy outcomes such as LBW and preterm labor in housewives compared to employed women. Thus, it is recommended that health personnel, especially midwives attempt to resolve these problems in housewives by raising their awareness and changing their attitude toward health through teaching them the importance of prenatal care and its effect on pregnancy outcomes (M, 2007). In the present study, there was no significant difference between pregnant women in mean daily working hours and standing hours in terms of placenta previa, placental abruption, reduced amniotic fluid, gestational age, or LBW. Cesarean section was 47.6% in women with less than 8 hours of daily standing, and 100% in those with more than 8 hours of standing. This may have been due to employment, higher education, and greater tendency toward elective cesarean. The study findings of Naseriasl showed a significant relationship between cesarean section and employment (Naseriasl, 2013).

In the present study, no relationship was found between lifting heavy objects and placenta abruption and placenta previa, mode of delivery or gestational age. Unlike other studies that found a significant relationship between physical activity and temporary contractions and preterm labor, our study revealed a significant relationship between physical activity and low birth weight (Niedhammer et al., 2009). Everyday occupational exposures are related to working hours, shift work, standing, lifting, and physical workload (Palmer et al., 2013). In theory, such common exposures could affect the outcomes of pregnancy. For example, disrupted circadian rhythms from shift working could trigger neuroendocrine changes that affect fetal growth and timing of parturition, while raised noradrenaline levels from heavy physical exertion could increase uterine contractility and risks of preterm labour (Chasan et al., 2007). The present study showed a significant relationship between lifting heavy objects and reduced amniotic fluid, but no other study has been done in this area. Most studies have conducted regarding the effect of employment on pregnancy outcomes are prospective, and showed the effects on birth weight, preterm labor, and intrauterine growth retardation. Some studies have examined short-term and even long-term future effects of employment on children (Bonzini et al., 2006; Palmer, 2013). A study showed that pregnant women exposed to chemicals and solvents at work had children with pulmonary wheezing two years after birth (Bajeux et al., 2014). Moreover, exposure of pregnant women to aromatics and preservatives in food industry led to giving birth to infants too small for gestational age (Langlois et al., 2014).

A study on Norwegian women investigated sick leave in terms of age and type of work. The results showed that working women increasingly delayed pregnancy for promotion, and pregnancy was more common in older age, but the number of sick leave days was higher in less than 20 year-old pregnant women compared to women older than 45 years of age. This may be due to the fact that younger women comprise greater proportion of working population. However, the relationship between the number of sick leave days and age reversed when type of job was controlled and older women took more days off due to sickness. Parity is also an important factor, and sickness days are high in women with multiple parities from any age group (Ariansen, 2014; Salihu, Myers, & August, 2012).

In another study, no relationship was found between psychological stresses of working mothers until 15 weeks (even with age and type of job controlled) and infant’s skeletal and cardiovascular abnormalities (Larsen et al., 2014; A. D. Larsen et al., 2013). Maternal exposure to allergens can afflict their children with asthma by the age of 7 years, disregarding child’s gender (Bajeux et al., 2014; Christensen et al., 2013).

Studies ruled out that psychosocial state of mothers can affect pregnancy outcomes. The majority of mothers with high anxiety during pregnancy give birth to irritable and LBW children with sleep disorders. Occupational stress can also cause undesirable pregnancy outcomes (Bonzini et al., 2009; Dabbaghi F. Sadeghi H, 2001). The results indicated that housewives usually have greater problems than working mothers, which may be due to the latter having higher education and social status. Considering risks due to preterm labor, placenta abruption, and reduced amniotic fluid, and their effect on the community and future generation, it is hoped that the present study results would contribute toward proper implementation of prenatal care, and also training and guidance of those at risk. It is hoped that the ultimate goal of healthy children born to healthy mothers is realized through applying the results of the present and other similar studies.

## 5. Conclusion

Our findings suggest that physical activity in pregnancy may predict reduced amniotic fluid, placental abruption and low birth weight. This is one of the few prospective studies on pregnancy outcomes that include working conditions. Thus proper postural habits and minimizing stress on the body and risk of injury maybe advantageous for pregnant women. However, employment alone does not predict pregnancy outcomes.

**Ethical Considerations**

The written consent letter was taken from health centers heads for study Also, The subjects announced her consents orally and completed questionnaires. Ethics Committee of Zahedan University of Medical Sciences has approved this research.
